# Surgical quality and long-term outcomes in advanced ovarian cancer: a population based registry study from Hamburg, Germany 2014–2024

**DOI:** 10.1007/s00404-026-08578-x

**Published:** 2026-10-29

**Authors:** Schultz Annemarie, Peters Frederik, Jaeger Anna, Schmalfeldt Barbara

**Affiliations:** 1 Hamburg Cancer Registry, Hamburg, Germany; 2 Department of Gynaecology, University Medical Centre Hamburg-Eppendorf, Hamburg, Germany

**Keywords:** Cytoreductive surgery, Survival analysis, Real-world evidence, Cancer registry, Complete resection, FIGO stage III/IV

## Abstract

**Purpose:**

To evaluate the association of surgical quality and survival in patients with advanced ovarian cancer in a real-world setting.

**Methods:**

This population-based registry study analysed FIGO stage III/IV ovarian cancer patients in Hamburg, Germany (2014–2024). Patients were categorized by surgical outcome (no macroscopic residuals vs. macroscopic residuals). Kaplan–Meier functions and Cox proportional hazards models estimated associations with survival outcomes.

**Results:**

The cohort comprised 576 patients (median age 65 years, median follow-up 57 months). No macroscopic residuals were achieved in 370 patients (64.2%). In multivariable analysis, no macroscopic residuals were associated with reduced mortality risk (HR 0.62, 95% CI: 0.42–0.90) and reduced progression risk (HR 0.68, 95% CI: 0.49–0.94). Advanced FIGO stage, poor performance status, and increasing age were independent predictors of worse outcomes.

**Conclusion:**

Achieving no macroscopic residuals remains a critical prognostic factor in advanced ovarian cancer. The rate of 64.2% demonstrates that optimal surgical outcomes are achievable in routine practice, though opportunities for improvement remain compared to specialized centres.

**Supplementary Information:**

The online version contains supplementary material available at https://doi.org/10.1007/s00404-026-08578-x.

## What does this study add to the clinical work?

**Table:** 

This population-based study confirms that achieving no macroscopic residuals remains the most strongly associated modifiable prognostic factor in advanced ovarian cancer, with significantly reduced mortality and progression risk. The complete resection rate of 64.2% in routine practice demonstrates achievable outcomes while identifying opportunities for quality improvement compared to specialized centres.	

## Introduction

Ovarian cancer remains the gynaecological malignancy with the highest mortality rate, despite continuous improvements in surgical and systemic treatment options over the years [1]. Although early-stage disease is highly curable, almost two thirds of the patients suffer from advanced stage of disease (stage III/IV) at initial diagnosis [1], with over 75% of patients with late-stage ovarian cancer dying of their disease [2–4]. This pattern is confirmed by current German registry data, showing that 77% of ovarian cancers are diagnosed in advanced FIGO stages III/IV, with a stark survival difference between early (86.1%) and advanced stages (37.2%) [5].

The quality of therapy is a key prognostic parameter in ovarian cancer, particularly residual tumour after debulking surgery [6]. Complete macroscopic resection has become the standard of care, with rates of 70–80% now achievable in specialized centres [7, 8].

Platinum-taxane-based chemotherapy is the second pillar in advanced ovarian cancer treatment [9].

Maintenance therapy with bevacizumab and/or PARP inhibitors prolongs progression-free survival [10–16], yet the combined impact of surgical quality and systemic therapy on outcomes remains incompletely understood. Most clinical trials evaluate these interventions separately, providing limited real-world evidence on their synergistic effects. Understanding how complete resection and optimal systemic therapy interact is crucial for treatment optimization and patient counselling, particularly as treatment patterns vary substantially across different healthcare settings and patient populations.

Recent German quality assurance data demonstrate improving outcomes in advanced ovarian cancer, with median progression-free survival increasing from 12.7 to 20.5 months between 2004 and 2016, attributed to improved surgical techniques and systemic therapy advances [17].

Comorbidities and frailty are substantially associated with reduced treatment adherence, with about one third of patients not receiving optimal therapy [17–19]. Histological subtype also influences prognosis, with high-grade serous cancers showing worse outcomes [20].

The German Cancer Society’s audit report indicates complete resection rates of 73.3% in certified centres [21, 22], comparable to specialized centres, such as the Mayo Clinic (75%) [21].

While the prognostic importance of complete cytoreduction is well-established, contemporary population-based evidence from routine clinical practice remains limited, particularly in the era of bevacizumab and PARP inhibitor availability, and for unselected patient populations including elderly and comorbid patients typically underrepresented in clinical trials. Hamburg’s metropolitan healthcare context, with universal insurance coverage and short distances to multiple certified centres, provides a setting in which achievable real-world surgical quality can be meaningfully assessed.

In this population-based study, we aimed to quantify the real-world survival outcomes associated with surgical quality in patients with advanced ovarian cancer. Our primary objective was to compare overall survival and progression-free survival between patients with no macroscopic residuals versus those with macroscopic residuals, while accounting for systemic therapy and other prognostic factors. Through this approach, we sought to evaluate the prognostic importance of complete cytoreduction in routine clinical practice and identify potential strategies for improving surgical outcomes.

## Methods

### Data source

The Hamburg Cancer Registry captures all cancer cases with mandatory reporting since 2013 and estimated completeness > 95% [23, 24].

### Patients

We included patients with advanced ovarian cancer diagnosis (Fédération Internationale de Gynécologie et d’Obstétrique (FIGO) stage III or IV [25]) between 2014 and 2024 registered in Hamburg, Germany, according to the International Classification of Diseases 10 German Modification (ICD10-GM) with code C56, C57.0, C57.4, C57.8, C48.1, C48.2, C48.8. We excluded cases with primary ovarian tumours before 2014 (based on data from the epidemiological registry spanning over the time 1990–2024), patients without residence in Hamburg, age at diagnosis < 18 or > 99, without surgery, or unknown systemic therapy. Only surgeries with curative intention were included. In cases of multiple tumour reports per patient, only the first case was considered. Patients with unknown residual disease status after surgery were not excluded, but were retained in the analytic cohort, with missing values addressed through multiple imputation.

### Study variables

The primary variable was surgical quality, defined as the presence or absence of macroscopic residual tumour after surgery, as documented in the German oncological base dataset (oBDS) [26]. Patients were grouped into no macroscopic residuals, defined as no grossly visible tumour remaining after surgery regardless of microscopic margin status, versus macroscopic residual disease, defined as any grossly visible tumour remaining after surgery. For FIGO stage IV disease, overall residual disease status was used, while for FIGO stage III, local residual disease status was prioritized. Surgical treatments were included if initiated within six months before or after diagnosis. The 6-month window was chosen based on observed treatment patterns in the registry, in which most patients initiated primary therapy within this timeframe. The window extends prior to diagnosis to account for cases in which surgery preceded the formal registry diagnosis date, as histological confirmation and reporting may follow the surgical procedure. In patients with multiple surgeries within the window, the date of the last surgery was used for further analysis, and the best achieved residual disease status across all surgeries was recorded. Primary cytoreductive surgery was defined as surgery performed prior to the initiation of first-line chemotherapy; interval cytoreductive surgery as surgery performed following neoadjuvant chemotherapy, with an interval of at least 29 days between diagnosis and first surgery. Systemic treatments were included if initiated within 6 months before or up to 12 months after diagnosis to capture first-line interventions. Only surgeries with curative treatment intention were included, as documented in the treatment intention field of the German oncological base dataset (oBDS), which is a mandatory reporting item for all surgical interventions. Systemic therapy was categorized as bevacizumab-containing versus other regimens. We focused on bevacizumab as it was consistently documented, unlike PARP inhibitors which were newly introduced during the study period [11, 13]. Variables included in this analysis were calendar year (grouped in 2014–2016, 2017–2019 and 2020–2024), FIGO stage (III or IV) age at diagnosis, histological subgroup according to the definitions of the World Health Organization [27] (endometroid carcinoma (ICD-O-3: 8380, 8474), low grade serous adenocarcinoma (ICD-O-3: 8460), high grade serous adenocarcinoma (ICD-O-3: 8461, 8441) and others (e.g. clear cell carcinoma (ICD-O-3: 8310) and mucinous (ICD-O-3: 8480)), performance status based on ECOG performance status (0 = fully active; 1 = restricted in physically strenuous activity, but ambulatory; > 1 = limited self-care capacity or bedridden) [28], grouped as 0–1 vs > 1. Where Karnofsky Performance Status was documented instead, values were converted to ECOG scores according to standard oBDS crosswalk criteria. Treatment in a certified gynaecological oncology centre was included as a binary variable.

Overall survival was defined as time from the date of the last surgery within six months after diagnosis to death from any cause within 8 years of follow-up, as cancer-specific mortality data were not consistently available in the registry. Progression-free survival was defined as time from the date of the last surgery within six months after diagnosis to disease progression or death from any cause, whichever occurred first, within 8 years of follow-up. Patients without events were censored at 8 years of follow-up or at the end of the study period (December 31, 2024).

### Statistical analysis

Descriptive statistics were presented using proportions for discrete variables and median for continuous variables. For estimating the impact of prognostic factors on PFS and OS, Kaplan–Meier functions with log rank tests and Cox proportional hazard regression models were applied. The proportional hazards assumption was assessed for all covariables using the global test and variable-specific scaled Schoenfeld residuals (cox.zph). Multiple imputation by chained equations with 20 iterations for 5 copies of the data were used to account for missing values in ECOG performance status, nodal status, and residual disease classification. The imputation model included FIGO stage, age at diagnosis, diagnosis period, histological subtype, ECOG performance status, treatment centre, surgical complications, interval between diagnosis and surgery, surgery type (primary vs. interval), nodal status, and residual disease status. Results pooled based on Rubin’s rules were presented as hazard ratios with 95% confidence intervals. We did not adjust for multiple testing.

Eight sensitivity analyses were performed to test the robustness of our findings: (1) complete case analysis excluding patients with missing data, (2) analysis restricted to patients treated in certified centres, (3) analysis excluding patients who did not achieve a follow-up of 12 months after diagnosis, (4) propensity score matched analysis using 1:1 nearest-neighbour matching without replacement, balancing baseline characteristics (FIGO stage, diagnosis period, age, histological subtype, ECOG performance status, and treatment centre) between surgical outcome groups, (5) three-year follow-up analysis, (6) analysis excluding patients aged ≥ 75 years, and (7) analysis excluding patients who died within 365 days after diagnosis, and (8) analysis restricted to patients with no documented neoadjuvant chemotherapy prior to surgery.

R version 4.3.1 was used to perform all analyses and plots (R Core Team, 2023, https://www.R-project.org/). We adhered to the Strengthening the Reporting of Observational Studies in Epidemiology (STROBE) for reporting our results [29].

## Results

### Patient characteristics

**Fig. 1:**
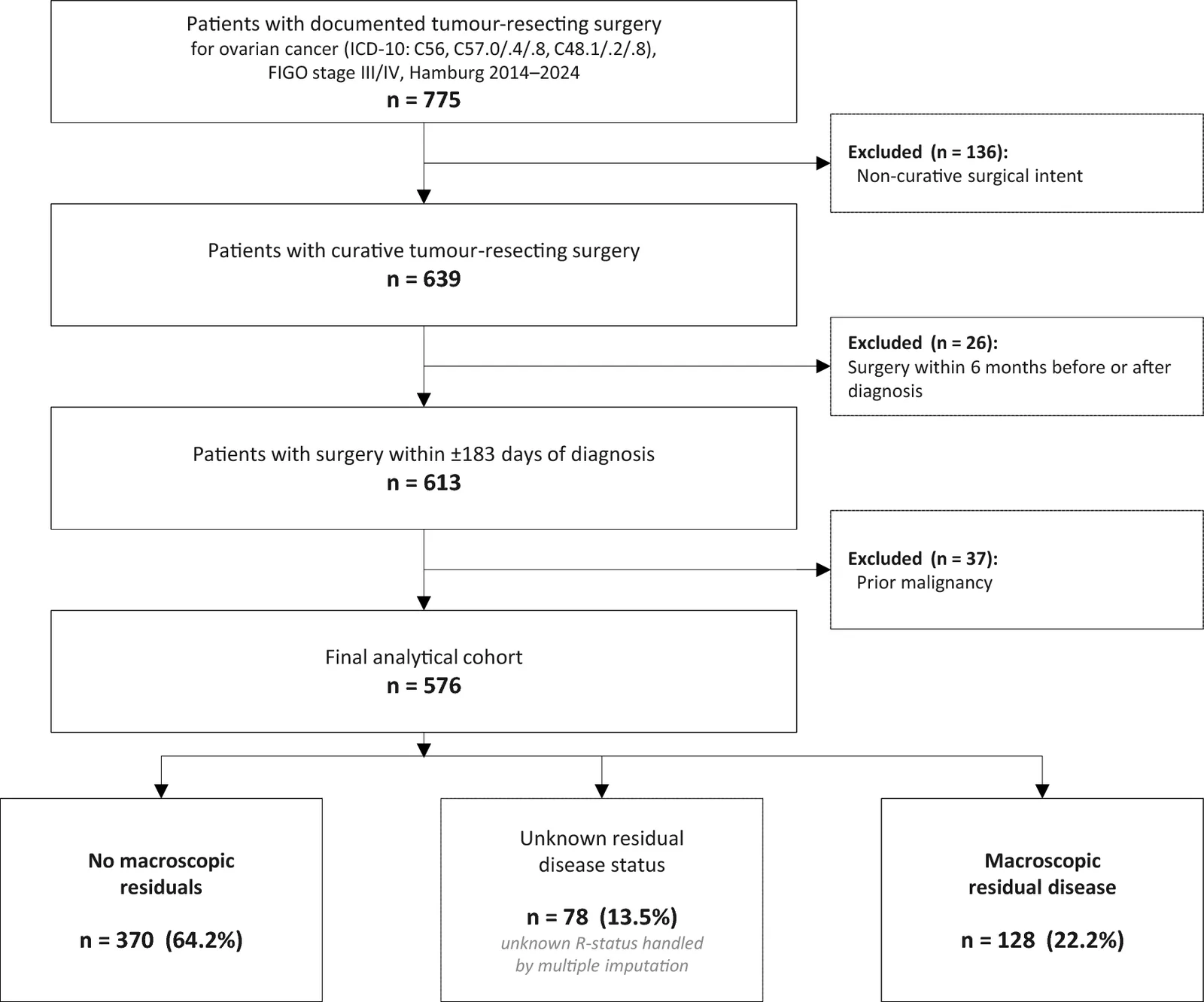
Patient flow diagram

A total of 576 patients were included (median age 65 years; median follow-up 57 months for OS and 55 months for PFS; Fig. 1, Table 1). High-grade serous histology (74.1) and FIGO stage III (65.8%) predominated. Within eight years, 295 patients (51.2) died and 353 (61.3%) experienced progression or death. No macroscopic residuals were achieved in 370 patients (64.2%), while 128 patients (22.2%) had macroscopic residual disease and 78 patients (13.5%) had unknown resection status. Among patients with no macroscopic residuals, 180 (48.6%) received bevacizumab-containing first-line therapy and 190 (51.4%) received platinum-taxane or other regimens without bevacizumab. In the macroscopic residual disease group, 66 (51.6%) received bevacizumab maintenance and 62 (48.4%) received other regimens.

**Table 1:** Baseline characteristics of patients with advanced ovarian cancer by surgical outcome

Characteristic	Overall (*n* = 576)	No macroscopic residuals (*n* = 370)	Macroscopic residuals (*n* = 128)	Unknown (*n* = 78)
Age at diagnosis (median, Q1, Q3)	65 (56, 75)	64 (56, 73)	68.5 (59, 78)	66 (54, 77)
FIGO Stage IV, N (%)	197 (34.2)	114 (30.8)	59 (46.1)	24 (30.8)
Chemotherapy, N (%)				
Carboplatin mono	35 (6.1)	20 (5.4)	9 (7.0)	6 (7.7)
Carboplatin + paclitaxel	397 (68.9)	266 (71.9)	88 (68.8)	43 (55.1)
None	144 (25.0)	84 (22.7)	31 (24.2)	29 (37.2)
Bevacizumab = Yes, N (%)	277 (48.1)	180 (48.6)	66 (51.6)	31 (39.7)
PARP inhibitor = Yes, N (%)	135 (23.4)	89 (24.1)	36 (28.1)	10 (12.8)
Neoadjuvant therapy = Yes, N (%)	64 (11.1)	40 (10.8)	13 (10.2)	11 (14.1)
Diagnosis period, N (%)				
2014–2016	96 (16.7)	46 (12.4)	19 (14.8)	31 (39.7)
2017–2019	178 (30.9)	126 (34.1)	29 (22.7)	23 (29.5)
2020–2024	302 (52.4)	198 (53.5)	80 (62.5)	24 (30.8)
ECOG Performance Status, N (%)				
0 or 1	326 (56.6)	225 (60.8)	73 (57.0)	28 (35.9)
> 1	60 (10.4)	32 (8.6)	22 (17.2)	6 (7.7)
Missing	190 (33.0)	113 (30.5)	33 (25.8)	44 (56.4)
Treatment in certified centre, N (%)	463 (80.4)	311 (84.1)	114 (89.1)	38 (48.7)
Histology, N (%)				
Endometrioid or low grade serous	48 (8.3)	31 (8.4)	5 (3.9)	12 (15.4)
Clear cell or mucinous	27 (4.7)	17 (4.6)	4 (3.1)	6 (7.7)
High grade serous	427 (74.1)	280 (75.7)	105 (82.0)	42 (53.8)
Other	74 (12.8)	42 (11.4)	14 (10.9)	18 (23.1)
Nodal state, N (%)				
Negative	274 (47.6)	177 (47.8)	67 (52.3)	30 (38.5)
Positive	258 (44.8)	177 (47.8)	50 (39.1)	31 (39.7)
Missing	44 (7.6)	16 (4.3)	11 (8.6)	17 (21.8)
Complications = Yes, N (%)	63 (10.9)	35 (9.5)	19 (14.8)	9 (11.5)
Primary surgery, N (%)	519 (90.1)	333 (90.0)	117 (91.4)	69 (88.5)

*ECOG* Eastern Cooperative Oncology Group, *FIGO* International Federation of Gynaecology and Obstetrics, *Q1* First quartile, *Q3* Third quartile

Baseline differences between groups included higher FIGO stage IV rates in the macroscopic residuals group (46.1% vs 30.8%) and older median age (69 vs 64 years). The rate of no macroscopic residuals (64.2%) reflects real-world treatment patterns.

### Unadjusted survival outcomes by treatment group

**Fig. 2:**
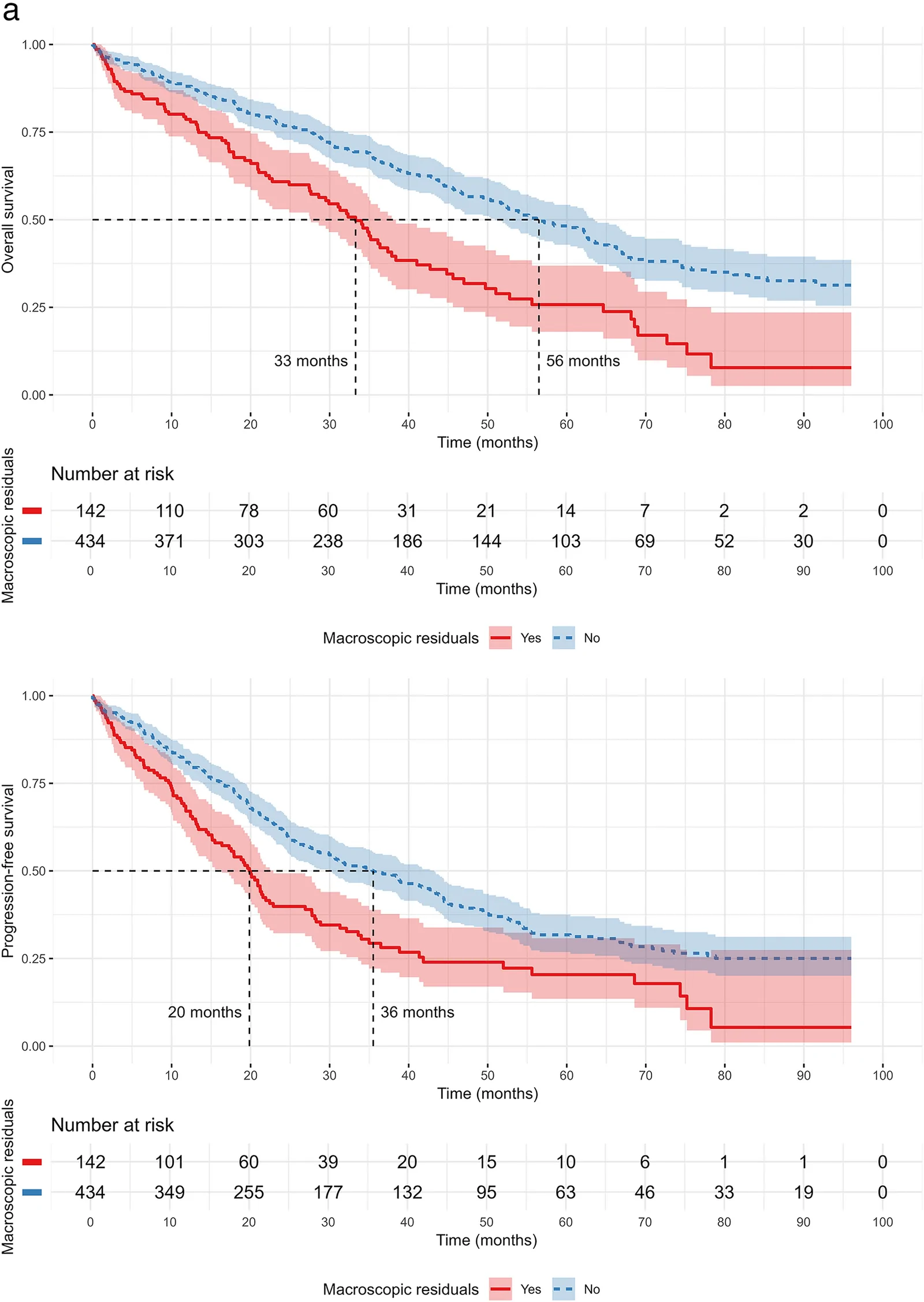

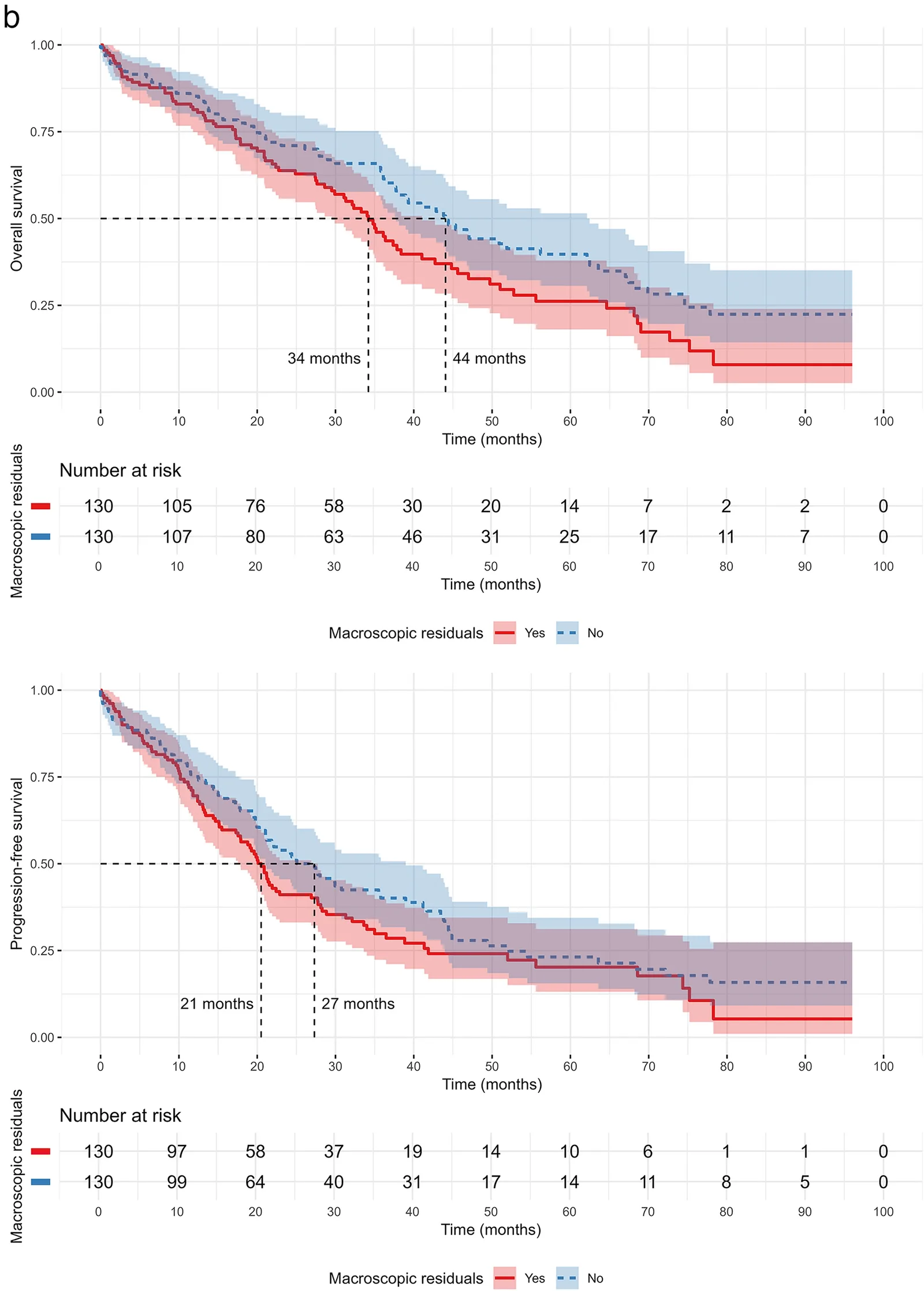
**A** Overall survival and progression-free survival of patients with advanced ovarian cancer by surgical outcome (*n* = 576) **B** Overall survival and progression-free survival of patients with advanced ovarian cancer by surgical outcome, after propensity score matching based on FIGO stage, diagnosis period, age, histological subtype, ECOG performance status, and treatment centre (*n* = 260)

Marked differences were observed in both overall survival and progression-free survival between surgical outcome groups (Fig. 2A). Patients with no macroscopic residuals demonstrated superior outcomes compared to those with macroscopic residual disease across both endpoints. Median overall survival was 56 months in the no macroscopic residuals group compared to 33 months in the macroscopic residual disease group. Median progression-free survival was 36 months in the no macroscopic residuals group versus 20 months in the macroscopic residual disease group (Fig. 2A).

Propensity score matching (*n* = 260, 130 pairs), no macroscopic residuals remained associated with improved overall survival (HR 0.72, 95% CI: 0.52–1.00) and progression-free survival (HR 0.84, 95% CI: 0.62–1.13), consistent in direction with the primary analysis, though not reaching statistical significance, likely reflecting the reduced statistical power of the matched subsample (Fig. 2B).

### Multivariable analysis of factors associated with overall survival

**Fig. 3:**
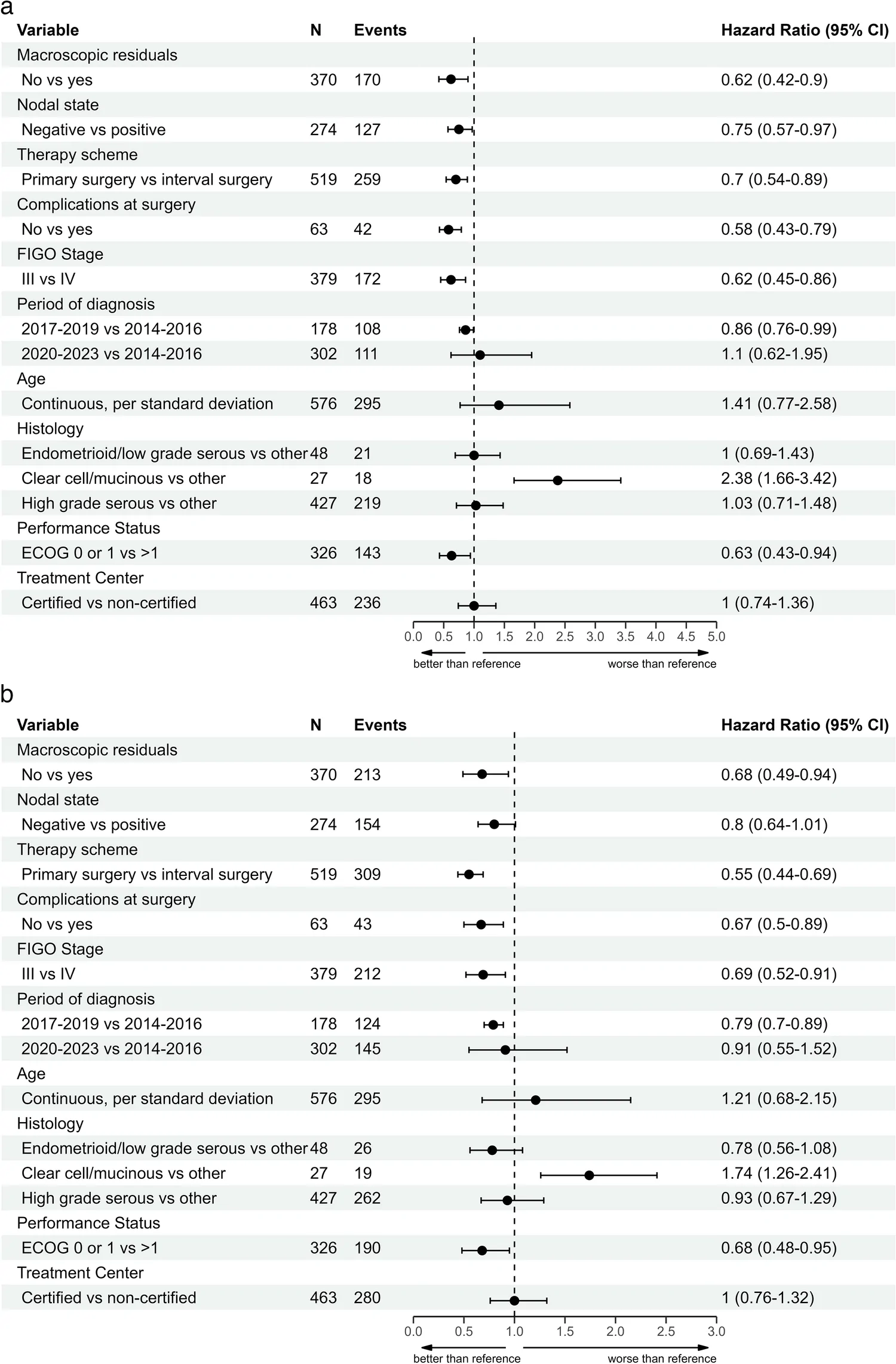
Multivariable analysis of factors associated with overall survival **A** and progression-free survival **B** in patients with advanced ovarian cancer

In multivariable analysis for overall survival (Fig. 3A), no macroscopic residuals were associated with a 38% reduced mortality risk compared to macroscopic residual disease (HR 0.62, 95% CI: 0.42–0.90). Among clinical factors, FIGO stage III was associated with a 38% reduced mortality risk compared to stage IV **(**HR 0.62, 95% CI: 0.45–0.86), and good performance status (ECOG 0–1) reduced mortality risk by 37% (HR 0.64, 95% CI: 0.43–0.94). Primary surgery was associated with a 30% reduced mortality risk compared to interval surgery (HR 0.70, 95% CI: 0.47–1.02), though it did not reach statistical significance.

### Multivariable analysis of factors associated with progression-free survival

In multivariable analysis for progression-free survival (Fig. 3B), no macroscopic residuals were associated with a 32% reduced risk of progression or death compared to macroscopic residual disease (HR 0.68, 95% CI: 0.49–0.94). FIGO stage III was associated with a 31% reduced risk compared to stage IV (HR 0.69, 95% CI: 0.54–0.87), and good performance status (ECOG 0–1) was associated with a 32% reduced risk (HR 0.68, 95% CI: 0.48–0.95). Primary surgery showed the strongest association with a 45% reduced risk compared to interval surgery (HR 0.55, 95% CI: 0.39–0.78).

The proportional hazards assumption was satisfied for the primary exposure, residual disease status, in both models (scaled Schoenfeld residuals: OS *p* = 0.785, PFS *p* = 0.161), with no systematic trend over time. The global test was significant for the overall survival model (*p* = 0.021). This reflected time-varying effects of age (*p* = 0.001), histology (*p* = 0.002), surgical complications (*p* = 0.005), and nodal status (*p* = 0.049), which are well-recognised in long-term observational cohorts and do not affect the estimate for the primary exposure. An accelerated failure time model with a Weibull distribution yielded concordant estimates for the primary exposure (OS HR 0.66, 95% CI 0.46–0.92; PFS HR 0.68, 95% CI 0.49–0.93), confirming robustness to this assumption.

### Sensitivity analyses

Eight sensitivity analyses confirmed robust associations (Supplementary Table S1). Complete case analysis (*n* = 336), certified centres only (*n* = 463), propensity score matching (*n* = 260), three-year follow-up, and excluding patients aged ≥ 75 years, and an analysis restricted to patients classified as undergoing primary cytoreductive surgery (no documented neoadjuvant chemotherapy prior to surgery; *n* ≈ 506) all showed consistent HRs between 0.51 and 0.72 for overall survival and 0.57–0.84 for progression-free survival.

## Discussion

This population-based study demonstrates that no macroscopic residuals (achieved in 64.2% of patients) were associated with 38% reduced mortality (HR 0.62) and 32% reduced progression risk (HR 0.68), consistent across all sensitivity analyses.

When comparing our survival outcomes to national reference data, our results suggest reasonable treatment quality despite the lower complete resection rate. While direct comparison with Robert Koch Institute cancer statistics 2019/2020 has limitations given our broader inclusion criteria (C56, C57.x, C48.x versus RKI’s C56 only), the reported relative 5-year survival rates for ovarian cancer of 42% for FIGO stage III and 21% for FIGO stage IV [30] provide useful context. Our survival patterns by resection status appear broadly consistent with these national benchmarks, suggesting that overall treatment quality in Hamburg achieves outcomes within expected ranges despite the documented challenges in achieving complete resection.

Our results strongly align with established evidence demonstrating the critical importance of optimal cytoreduction in advanced ovarian cancer [6]. The significant survival benefits observed across both overall survival and progression-free survival reinforce that surgical quality remains the most strongly associated modifiable prognostic factor in this disease [2–4]. The consistency of our findings across multiple sensitivity analyses, including propensity score matching with 260 patients, complete case analysis with 336 patients, and subgroup analyses, supports the robustness of the association between no macroscopic residuals and improved outcomes. As an observational analysis restricted to measured variables, however, residual confounding cannot be excluded, in particular from factors not captured in the registry, such as comorbidity burden, tumour burden (e.g., peritoneal cancer index), surgical complexity, and surgeon or institutional experience.

Our rate of no macroscopic residuals (64.2%) differs from quality assurance data reporting rates of 73.3–85% [17, 23]. However, these rates typically include residual disease < 1 cm, which differs from the strict criterion of no grossly visible residual tumour. The differences may reflect selection bias in certification data, broader patient inclusion in our registry, and incomplete documentation. Standardized documentation of surgical outcomes is essential for accurate quality assessment [21, 31].

While the prognostic importance of optimal cytoreduction is well-established, our study contributes important population-based evidence on treatment patterns and outcomes in routine clinical practice. Unlike highly selected clinical trial populations, our cohort reflects the full spectrum of patients treated in a metropolitan cancer care system, including elderly patients and those with comorbidities who are typically underrepresented in interventional studies. The rate of no macroscopic residuals (64.2%) documented here provides crucial insights into real-world treatment gaps and identifies opportunities for quality improvement initiatives. Whether this reflects patient selection (e.g., more patients deemed unsuitable for aggressive cytoreduction), surgical practice patterns, or documentation issues warrants further investigation.

The high rate of missing ECOG (33.0%) highlights challenges in routine documentation, emphasizing the need for improved surgical outcome reporting as recommended by clinical guidelines [6]. Regarding the median age at initial diagnosis (65 years), FIGO stage distribution (65.8% FIGO III, 34.2% FIGO IV) and histological subtype (74.1% high-grade serous) our results are comparable to previous studies [31, 32].

Interestingly, the percentage of patients treated in experienced, certified gynaecological oncology centres in our study was high with 80.4% compared to other studies. This could be attributed to the fact that the study region Hamburg represents a metropolitan area with short distances to specialised care and a generally high coverage rate of health services. Notably, approximately 25% of patients did not receive documented systemic chemotherapy, a proportion higher than expected based on guideline recommendations. This may reflect incomplete documentation, contraindications in frail patients, or patient refusal, and warrants further investigation.

Sensitivity analysis excluding patients ≥ 75 years showed particularly strong associations (HR 0.51 for OS, HR 0.57 for PFS), suggesting surgical quality may be especially strongly associated with outcomes in younger patients [4, 19, 33]. The propensity score matched analysis yielded results consistent in direction with the primary analysis; the absence of statistical significance likely reflects the reduced power of the matched subsample of 260 patients. These findings underscore the importance of individualizing treatment decisions based on patient condition and functional status.

### Limitations and strengths

Several important limitations warrant acknowledgment. First, we lacked information on BRCA1/2 mutations and homologous recombination deficiency status. Second, PARP inhibitor documentation was incomplete. Third, the number of patients with macroscopic residual disease (*n* = 128) limits statistical power.

Fourth, as an observational study, unmeasured confounders including surgeon experience and comorbidity profiles may influence outcomes. Although all patients had undergone surgery with curative intent, detailed comorbidity data were not available in the registry. Prior malignancies, ECOG performance status, and surgical complications served as partial proxies for patient frailty; however, surgeon experience and institutional case volume were not captured and may have independently influenced outcomes. Furthermore, as clinical cancer registration in Hamburg was established in 2013, documentation completeness for surgical procedures and clinical variables may have been lower in the earlier years of the study period, potentially introducing information bias. Additionally, competing risks analysis for non-cancer mortality was not performed, which may be relevant given the proportion of elderly patients. Multiple sensitivity analyses and two survival endpoints were evaluated without adjustment for multiple testing. Results should, therefore, be interpreted in the context of the overall consistency of findings across analyses. Finally, the high proportion of patients treated in certified gynaecological oncology centres (80.4%) reflects Hamburg’s specific metropolitan infrastructure and may not be representative of broader national or international practice patterns. This contextual characteristic should be considered when generalising these findings to other settings.

Notable strengths include the population-based design capturing real-world practice, high registry completeness (> 95%) [24], assessment of both PFS and OS, multiple sensitivity analyses including propensity score matching (*n* = 260), and use of multiple imputation to mitigate bias from missing data.

## Conclusion

This population-based study demonstrates that achieving no macroscopic residuals remains strongly associated with improved survival outcomes in advanced ovarian cancer. No macroscopic residuals were associated with significantly reduced mortality risk and reduced risk of progression or death, with consistent findings confirmed across multiple sensitivity analyses. The rate of achieving no macroscopic residuals in this population-based cohort demonstrates that optimal surgical outcomes are attainable in routine practice, though opportunities for further improvement remain when compared to specialized centres.

The consistency of surgical quality effects across all analytical approaches reinforces that avoiding macroscopic residual disease remains the most strongly associated modifiable prognostic factor in advanced ovarian cancer. These findings emphasize the continued importance of surgical quality as strongly associated with outcomes and support efforts to centralize care in high-volume centres with expertise in complex cytoreductive surgery. Additionally, improved documentation of surgical outcomes in routine practice is essential for accurate quality assessment and monitoring of treatment improvements over time.

## Supplementary Information

Below is the link to the electronic supplementary material.

Supplementary file1 (DOCX 16 KB)

## Data Availability

The datasets generated and analysed during the current study are not publicly available due to privacy regulations governing cancer registry data, but are available from the Hamburg Cancer Registry upon reasonable request and with appropriate ethical approvals.
