# Supplementary material for: Surgical quality and long-term outcomes in advanced ovarian cancer: a population based registry study from Hamburg, Germany 2014–2024
Source: Arch Gynecol Obstet. 2026 Oct 29;313(1):302. doi: 10.1007/s00404-026-08578-x (PMC13719812; doi:10.1007/s00404-026-08578-x)
Supplement: Supplementary file 1 [file 404_2026_8578_MOESM1_ESM.docx]

**Supplementary Tables**

**Table S1: Sensitivity Analyses for Treatment Effects on Survival Outcomes** Results from sensitivity analyses examining the robustness of treatment effects. Treatment groups are surgical therapy (No macroscopic residuals vs Macroscopic residuals as reference). All models adjusted for FIGO stage, diagnosis period, age, histological subtype, ECOG performance status, and treatment centre (where applicable). HR = Hazard ratio; CI = Confidence interval.

| **A) Complete case analysis (n = 336)** | | |
| --- | --- | --- |
| **Treatment** | **Overall survival HR (95% CI)** | **Progression-free Survival HR (95% CI)** |
| No macroscopic residuals vs Macroscopic residuals | 0.60 (0.42–0.85) | 0.68 (0.50–0.94) |
| **B) Certified centres only (n = 463)** | | |
| **Treatment** | **Overall survival HR (95% CI)** | **Progression-free Survival HR (95% CI)** |
| No macroscopic residuals vs Macroscopic residuals | 0.62 (0.44–0.87) | 0.68 (0.50–0.93) |
| **C) Excluding early deaths and progression within 365 days (n ≈ 480)** | | |
| **Treatment** | **Overall survival HR (95% CI)** | **Progression-free Survival HR (95% CI)** |
| No macroscopic residuals vs Macroscopic residuals | 0.58 (0.40–0.83) | 0.67 (0.48–0.93) |
| **D) Propensity score matched analysis (n = 260)** | | |
| **Treatment** | **Overall survival HR (95% CI)** | **Progression-free Survival HR (95% CI)** |
| No macroscopic residuals vs Macroscopic residuals | 0.72 (0.52–1.00) | 0.84 (0.62–1.13) |
| **E) Three years of follow-up (n = 576)** | | |
| **Treatment** | **Overall survival HR (95% CI)** | **Progression-free Survival HR (95% CI)** |
| No macroscopic residuals vs Macroscopic residuals | 0.63 (0.45–0.87) | 0.65 (0.49–0.85) |
| **F) Excluding patients age 75 years and older (n ≈ 420)** | | |
| **Treatment** | **Overall survival HR (95% CI)** | **Progression-free Survival HR (95% CI)** |
| No macroscopic residuals vs Macroscopic residuals | 0.51 (0.33–0.79) | 0.57 (0.39–0.83) |
| **G) Early death analysis - Excluding patients who died within 365 days (n ≈ 495)** | | |
| **Treatment** | **Overall survival HR (95% CI)** | **Progression-free Survival HR (95% CI)** |
| No macroscopic residuals vs Macroscopic residuals | 0.66 (0.40–1.08) | 0.62 (0.41–0.94) |
| **H) Patients classified as undergoing primary cytoreductive surgery (n ≈ 506)** | | |
| **Treatment** | **Overall survival HR (95% CI)** | **Progression-free Survival HR (95% CI)** |
| No macroscopic residuals vs Macroscopic residuals | 0.64 (0.46–0.90) | 0.68 (0.50–0.92) |
| **Note:** HR <1.0 indicates better outcomes for no macroscopic residuals compared to macroscopic residuals. | | |

**Table S2: Balance of variables before propensity score matching**

| **Characteristic** | **No macroscopic residuals (n=434)** | **Macroscopic residuals (n=142)** |
| --- | --- | --- |
| FIGO stage III, n (%) | 304 (70.0) | 75 (52.8) |
| 2014–2016 | 69 (15.9) | 27 (19.0) |
| 2017–2019 | 147 (33.9) | 31 (21.8) |
| 2020–2024 | 218 (50.2) | 84 (59.2) |
| Treatment in certified centre, n (%) | 344 (79.3) | 119 (83.8) |
| ECOG status 0–1, n (%) | 375 (86.4) | 103 (72.5) |
| Nodal status negative, n (%) | 211 (48.6) | 83 (58.5) |
| Primary surgery, n (%) | 388 (89.4) | 131 (92.3) |
| No complications, n (%) | 393 (90.6) | 120 (84.5) |

**Table S3: Balance of variables after propensity score matching**

| **Characteristic** | **No macroscopic residuals (n=130)** | **Macroscopic residuals (n=130)** |
| --- | --- | --- |
| FIGO stage III, n (%) | 76 (58.5) | 70 (53.8) |
| 2014–2016 | 31 (23.8) | 24 (18.5) |
| 2017–2019 | 24 (18.5) | 31 (23.8) |
| 2020–2024 | 75 (57.7) | 75 (57.7) |
| Treatment in certified centre, n (%) | 112 (86.2) | 109 (83.8) |
| ECOG status 0–1, n (%) | 103 (79.2) | 99 (76.2) |
| Nodal status negative, n (%) | 74 (56.9) | 75 (57.7) |
| Primary surgery, n (%) | 120 (92.3) | 119 (91.5) |
| No complications, n (%) | 108 (83.1) | 111 (85.4) |
